# Can 3D T1 Post-Contrast MRI in A Radiomics-Machine Learning Model Distinguish Infective from Neoplastic Ring-Enhancing Brain Lesions? An Exploratory Study

**DOI:** 10.3390/diagnostics16060926

**Published:** 2026-03-20

**Authors:** Edwin Chong Yu Sng, Minh Bao Kha, Min Jia Wong, Nicholas Kuan Hsien Lee, Jonathan Cheng Yao Goh, So Jeong Park, Darren Cheng Han Teo, Wei Ming Chua, May Yi Shan Lim, Septian Hartono, Lester Chee Hoe Lee, Candice Yuen Yue Chan, Hwee Kuan Lee, Ling Ling Chan

**Affiliations:** 1 Department of Infectious Diseases, Changi General Hospital, 2 Simei Street 3, Singapore 529889, Singapore; 2 Duke-NUS Medical School, 8 College Rd., Singapore 169857, Singapore; 3 Bioinformatics Institute, A*STAR, 30 Biopolis St., Matrix, Singapore 138671, Singapore; bao.km210098@sis.hust.edu.vn (M.B.K.);; 4 School of Information and Communications Technology, Hanoi University of Science and Technology, No. 1 Dai Co Viet, Hanoi, Vietnam; 5 Department of Neuroradiology, Singapore General Hospital, 31 Third Hospital Ave., Singapore 168753, Singapore; 6 School of Computing, National University of Singapore, COM1, 13, Computing Dr., Singapore 117417, Singapore; 7 Department of Infectious Diseases, Sengkang General Hospital, 110 Sengkang E Wy, Singapore 544886, Singapore; 8 Department of Neurosurgery, Changi General Hospital, 2 Simei Street 3, Singapore 529889, Singapore; 9 Department of Infectious Diseases, Singapore General Hospital, 31 Third Hospital Ave., Singapore 168753, Singapore; 10 School of Biological Sciences, Nanyang Technological University, 60 Nanyang Dr., Singapore 637551, Singapore; 11 International Research Laboratory on Artificial Intelligence, 1 Fusionopolis Way, #21-01 Connexis (South Tower), Singapore 138632, Singapore; 12 A* Centre for Frontier AI Research (CFAR), 1 Fusionopolis Way #16-16 Connexis (North Tower), Singapore 138632, Singapore

**Keywords:** ring-enhancing brain lesions, radiomics, machine learning, brain abscess, brain metastasis

## Abstract

**Background/Objectives**: Rapid and accurate classification of ring-enhancing brain lesions (REBLs) into infection or neoplasm is key to clinical triaging for expedited diagnostics in the former to enhance treatment outcomes, especially in the immunocompromised patients. High-resolution three-dimensional (3D) T1 post-contrast (T1+C) MRI provides high-dimensional volumetric data for radiomics analysis. While radiomics is useful in brain neoplasm characterization, its utility in central nervous system infection remains under-explored. In this exploratory study, we aim to determine if a radiomics-machine learning model, based solely on a 3D T1+C MRI dataset, can distinguish infective from neoplastic REBLs. **Methods**: 92 patients (infection, n = 26; neoplasm, n = 66) with 402 REBLs, who fulfilled criteria for “definite” or “probable” infective or neoplastic REBLs, were identified from scans performed at our hospital over four years and formed the training/validation dataset. All REBLs were manually annotated on T1+C MRI images under radiological supervision. In total, 1197 radiomics features were extracted, feature selection performed using mutual information, and nine machine learning classifiers applied to assess patient-level infection vs. neoplasm classification performance. End-to-end 2D CNN baselines and hybrid radiomics–CNN configurations were additionally evaluated under the same protocol for comparative benchmarking. Model performance was tested on an external holdout dataset of 57 patients (infection, n = 25; neoplasm, n = 32) with 454 REBLs from another hospital. **Results**: The Multi-layer Perceptron (MLP) model using the Original + LoG + Wavelet feature group demonstrated superior performance. In the cross-validation cohort, it achieved a mean AUC of 0.80 ± 0.02, sensitivity of 0.83 ± 0.09, specificity of 0.77 ± 0.08, and balanced accuracy of 0.80 ± 0.02. On external holdout data, the same configuration showed stable and sustainable performance with an AUC of 0.84, sensitivity of 0.84, specificity of 0.75, and balanced accuracy of 0.80. **Conclusions**: Our radiomics-machine learning model, based solely on a high-resolution 3D T1+C dataset, shows potential for distinguishing infective REBLs from neoplastic REBLs. Further study, with additional MR sequences and clinical data in a multimodal MRI radiomics-machine learning model, is warranted.

## 1. Introduction

**Figure 1:**
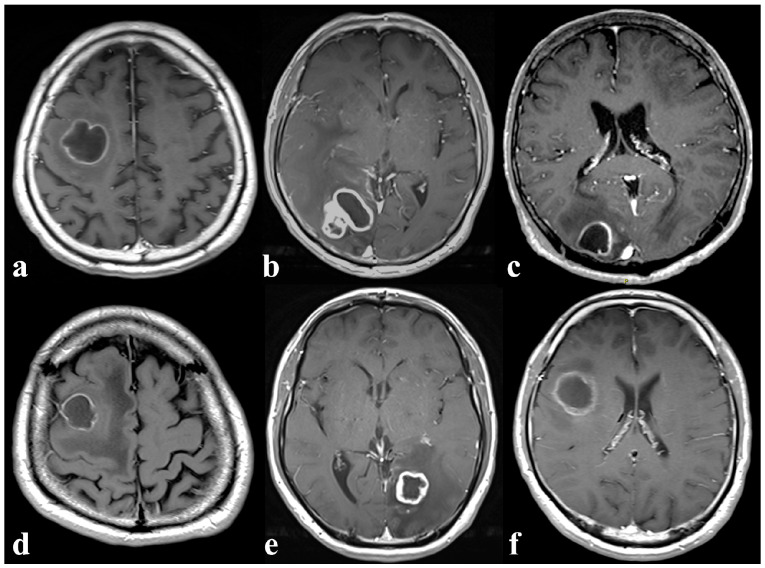
Axial post-contrast T1-weighted MRI images demonstrating ring-enhancing brain lesions due to (**a**) pyogenic brain abscess, (**b**) tuberculosis, (**c**) nocardiosis, (**d**) lung metastasis, (**e**) renal metastasis and (**f**) glioblastoma, as confirmed by clinico-microbiological and/or histopathological data [original image].

Contrast-enhanced T1-weighted (T1+C) magnetic resonance imaging (MRI) is routine in Radiology protocols for detection of suspected focal brain lesions. A ring-enhancing brain lesion (REBL) is a radiological abnormality describing a hypointense lesion surrounded by a bright rim of contrast enhancement from blood–brain barrier disruption. REBLs may be infective (e.g., pyogenic abscess), or neoplastic (e.g., metastasis) in origin [1]. In tertiary referral centers with large numbers of patients immunocompromised by underlying disease or treatment, REBLs may pose a significant diagnostic challenge as opportunistic infections (e.g., nocardiosis, toxoplasmosis) add to the list of differential diagnoses (Figure 1). Because patients with infection can rapidly deteriorate with high morbidity and mortality [2], rapid and accurate distinction is crucial to guide subsequent diagnostic evaluation and treatment, which are vastly different between infection and neoplasm. When there is diagnostic uncertainty, empirical antibiotics are often administered and brain biopsies may be performed unnecessarily, potentially resulting in avoidable side effects and complications such as antimicrobial resistance or devastating neurosurgical sequelae.

Notwithstanding, limitations to current diagnostic approaches exist [1,2,3,4,5,6]. The classic triad of headache, fever, and focal neurologic deficit is present in less than a quarter of patients on admission [2,7,8]. There is significant overlap in inflammatory markers such as white cell count and C-reactive protein between patients with infection and those with neoplasm [1], and crucial microbiological investigations such as blood cultures are usually unavailable at presentation. While certain neuroimaging features may aid in distinguishing underlying pathological processes [3,4], there are many exceptions to the rule. For example, while satellite lesions are more characteristic of abscesses than neoplasms [9], these are insensitive markers [10]. On diffusion-weighted imaging (DWI), cavities of abscesses classically exhibit marked hyperintensity from restricted diffusion of contents, while those of cystic/necrotic neoplasms exhibit hypointensity, but reports of abscesses with DWI-hypointense cavities [11,12,13] and neoplasms with DWI-hyperintense cavities abound [14,15,16]. The subjective nature of radiological assessment, combined with factors such as reader experience, fatigue and high workload, can compromise diagnostic accuracy [6]. Yet, accurate radiological classification into infection or neoplasm is key to triaging for immediate clinical decision-making on the diagnostic and management pathways, including whether expedited drainage or more elective/facultative biopsy of the brain lesion should be undertaken. Direct sampling of the brain lesion offers the highest diagnostic yield and is imperative for establishing the exact microbiological/histological diagnosis for definitive antimicrobial or oncological treatment. However, it may be associated with untenable risks of neurological deficits and hemorrhage [5], and patient comorbidities may also preclude neurosurgery under general anesthesia. An automated 24/7 objective and accurate imaging-based classification tool could help guide the final clinical decision for an elective or expedited high-risk biopsy following patient stabilization.

High-resolution three-dimensional (3D) T1+C scans are widely used today due to enhanced small lesion detection, detailed structural characterization, multiplanar capabilities and increasingly shortened scan times [17,18,19,20]. Radiomics coupled with machine learning (ML) algorithms are powerful analytical techniques that use high-dimensional quantitative data from radiological images for model building and clinical prediction [21,22]. Specifically, uniform isotropic voxel sizes in medical images enhance feature stability and reproducibility in radiomics-ML models [23,24]. These have demonstrated good potential in brain neoplasm characterization [25,26,27,28], yet their utility in distinguishing infection from neoplasm remains largely under-explored [29,30,31,32]. The scanty radiomics studies in the literature, summarized in Supplementary Table S1, were trained on 2D datasets, based on 4–6 mm thick T1+C and T2 fluid-attenuated inversion recovery (FLAIR) [29,30] or DWI MRI alone [32], and compared brain abscess versus a specific neoplastic etiology [29,30,32].

We hypothesize that 3D T1+C radiomics is valuable in distinguishing infective from neoplastic REBLs as 3D MRI acquisitions offer isotropic voxels that capture lesion morphology and spatial heterogeneity more accurately, providing richer volumetric information for radiomic feature extraction than conventional 2D imaging [18,20]. Leveraging this advantage, in this exploratory study, we aim to determine if a radiomics-ML model based solely on a 3D T1+C dataset can distinguish infective from neoplastic REBLs, using retrospective data accrued from two tertiary hospitals in our healthcare system.

## 2. Materials and Methods

### 2.1. Clinical Datasets

This retrospective study was approved by our centralized Institutional Review Board. Waiver of informed consent was granted. Our hospital, the largest tertiary referral hospital in our country, sees a high volume of immunocompromised patients ranging from those with chronic conditions such as diabetes mellitus, end-stage renal failure, autoimmune diseases, cancers and human immunodeficiency virus (HIV) infection to solid organ/hematopoietic stem cell transplant recipients. Radiological reports of all patients who underwent computed tomography (CT) or MRI brain scans between 1 November 2013 and 31 October 2017 were filtered for search terms indicative of REBLs (Supplementary Data S1). Two board-certified infectious disease physicians independently reviewed the electronic medical records of the identified patients to verify the final diagnoses. Diagnoses were defined as “definite” if a pathogen or a neoplasm was detected on brain tissue or cerebrospinal fluid (CSF) through cultures, histology, antigen or molecular testing, and both clinical presentation and treatment response were consistent with the diagnosis; and “probable” if a pathogen or a neoplasm was detected on blood or extracranial tissue, and both clinical presentation and treatment response were consistent with the diagnosis. Only patients whose diagnoses fulfilled criteria for either “definite” or “probable” and had a contrast-enhanced brain MRI study at initial presentation were included and formed our training/validation dataset. Our external holdout dataset came from another tertiary hospital in our healthcare system. Applying the same methodology, an infectious diseases physician reviewed the cases that were identified from scans performed between 1 December 2021 and 30 November 2024 from this hospital (data prior to 1 December 2021 were unavailable at point of study). Contrast-enhanced MRI brain study at presentation of patients’ whose diagnoses fulfilled criteria for either “definite” or “probable” formed the external holdout dataset.

All patients had clinical brain MRI performed on either Siemens Magnetom Avanto 1.5T or Skyra 3T scanners (Siemens Healthineers, Erlangen, Germany). Each study included two T1+C scans, (1) 2D axial spin echo (SE) and (2) 3D coronal gradient echo (GRE) sequence, acquired with the acquisition parameters displayed in Table 1.

### 2.2. Image Annotation and Pre-Processing

**Figure 2:**
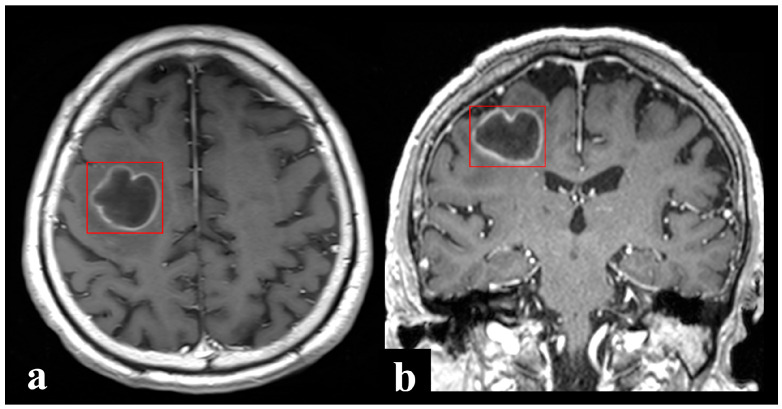
Depiction of bound box annotations of a right frontal ring-enhancing pyogenic brain abscess on (**a**) axial 2D and (**b**) coronal 3D post-contrast T1-weighted MRI images. [original image].

The REBLs were manually annotated by a research assistant and final-year medical students using the XNAT software (v1.8.8) under the direct supervision of board-certified neuroradiologists. XNAT is an open source imaging informatics platform that facilitates common management, productivity, and quality assurance tasks in imaging-based research. Each discrete REBL identified on 3D scans was annotated and sought for annotation on 2D axial scans (Figure 2). Bounding box annotations were made on each contiguous section to include the entire enhancing margins of the REBL. In this way, surrounding imaging context (vasogenic edema or non-enhancing lesional margins beyond the ring enhancement, e.g., in infiltrative primary brain tumors) which may contribute meaningfully to decision-making were also included, albeit incompletely. Satellite and multilocular lesions which could not be separated from the primary REBL/dominant lobule were included in the same bounding box. Related nodular or solid enhancing lesions (without central necrosis or cystic change) and meningeal involvement, when present, were also annotated. Detailed step-by-step annotation protocol is provided in Supplementary Data S2. All T1+C scans were uniformly resampled to 2mm isotropic voxels before radiomic feature extraction from the annotated bounding boxes.

### 2.3. Radiomics Feature Extraction

Quantitative 3D MRI features were automatically extracted using the PyRadiomics package (v3.0.1) [33] and implemented in Python (v3.9.10; https://python.org). To ensure consistency in signal intensity range across both the training-validation and test sets, voxel intensities of each MRI image were standardized using z-score normalization, and the normalized values were scaled by a factor of 100. Since normalization resulted in values below the mean (i.e., negative values), a voxel array shift of 300 (equivalent to 3 standard deviations scaled) was applied to keep most of the voxel intensities positive. Also, the bin width was set to 5 to discretize intensity values into a stable range. The extracted radiomic features were organized into three categories:

1. 3D shape-based features: These quantify the geometrical properties, including volume, surface area, sphericity, and various measures of elongation and flatness, hence providing insights into morphological characteristics.
2. Intensity-based (histogram) features: These describe the distribution of intensities with the annotated volume of interests, without considering their spatial relationships.
3. Textural-based features: These characterized the spatial arrangement and relationships of pixel values, capturing patterns and heterogeneity. The features include Grey-Level Co-occurrence Matrix (GLCM), Grey-Level Run Length Matrix (GLRLM), Grey-Level Size Zone Matrix (GLSZM), Grey-Level Dependence Matrix (GLDM), and Neighboring Grey Tone Difference Matrix (NGTDM). For this analysis, textural patterns were computed using parameters with a voxel distance of δ=1 across 13 angles (26-connectivity) in 3D space.

To enhance the feature space and capture multi-scale textural information, two types of filters were applied to patient-based imaging data:

1. Laplacian of Gaussian (LoG) filter: This operator accentuates regions of rapid intensity change within an image. LoG filters were applied with varying sigma values to capture image features at multiple spatial scales, ranging from coarse-grained textures (large sigma) to fine-grained textures (small sigma). Specifically, four sigma values (2.0, 3.0, 4.0, and 5.0 mm) were selected to encompass a range of spatial scales relevant to the structures of interest in medical imaging.
2. Wavelet decomposition filter: The Coifman Wavelet (Coiflet) with one vanishing moment was used as the mother filter for transformation. This decomposition produced eight combinations of wavelet coefficients: LLL, LLH, LHL, HLL, HHL, HLH, LHH, HHH. These transformations facilitated the extraction of directional and frequency-specific textural patterns, enabling a more comprehensive representation of multi-scale textural information.

### 2.4. Feature Selection Using Mutual Information

Feature selection is crucial to reduce model complexity, improve interpretability, and enhance model performance [34]. In this study, mutual information (MI) was employed as the feature selection technique to identify the most relevant features for classifying REBLs. MI measures the statistical dependence between the feature set $X=\{x_{1},x_{2},\cdots ,x_{n}\}$ and the target labels $Y=\{y_{1},y_{2},\cdots ,y_{n}\}$, where n is the number of the samples. Given a known joint distribution $P_{XY}(x_{i},y_{j})$ of two discrete random variables from groups X and Y, the marginal probabilities $P_{X}\left(x_{i}\right)$ and $P_{Y}\left(y_{i}\right)$ are calculated as follows:

$$P_{X}\left(x_{i}\right)=\sum_{j=1}^{n}P_{XY}\left(x_{i},y_{j}\right),P_{Y}\left(y_{i}\right)=\sum_{j=1}^{n}P_{XY}(x_{j},y_{i})$$

For a pair of random variables X and Y, the MI score I(X;Y) is defined as [35]

$$I\left(X;Y\right)=\sum_{i=1}^{n}\sum_{j=1}^{n}P_{XY}\left(x_{i},y_{j}\right)log\left(\frac{P_{XY}(x_{i},y_{j})}{P_{X}\left(x_{i}\right)P_{Y}\left(y_{j}\right)}\right)$$

Those features with an MI score greater than 0.12 were retained for the subsequent learning process to ensure their relevance in classification tasks. This threshold was selected empirically on the training/validation cohort by evaluating a range of MI cutoffs, and the chosen value retained an informative subset of radiomic features while filtering out low-relevance features, thereby reducing overfitting risk.

### 2.5. Machine Learning Classification

Nine different ML algorithms were applied to classify the extracted radiomics features, with the aim of identifying the most effective model for distinguishing infective REBLs from neoplastic REBLs. The selected classifiers represent common and conceptually diverse ML approaches, including distance-based, tree-based, boosting, and neural network methods, to provide a fair and comprehensive performance comparison. These were configured as follows:

1. Linear Regression (LR): 2000 iterations, L2 penalty, lbfgs solver.
2. Quadratic Discriminant Analysis (QDA): no interactions, reg_param = 0.
3. K-Nearest Neighbors (KNN): number of neighbors k=5, Euclidean distance metric, uniform weighting.
4. Decision Tree (DT): Gini impurity, best splitter, max depth = None.
5. Random Forest (RF): n_estimators = 100, Gini impurity, bootstrap sampling.
6. Support Vector Machine (SVM): RBF kernel, regularization parameter C = 1.0, probability estimates enabled.
7. AdaBoost: utilized the Decision Tree with max depth 1 as the base estimator, n_estimators = 50, learning rate = 1.0, SAMME.R boosting algorithm.
8. XGBoost: n_estimators = 100, with the default setting of gbtree booster, max depth = 6, learning rate = 0.3.
9. Multi-layer Perceptron (MLP): Two hidden layers with 32 and 16 neurons, respectively; ReLU activation function; Adam optimizer; batch size = 4; learning rate = 0.001. For each feature group, the model is trained for 100 iterations, and the parameters yielding the highest AUC_mean score on the validation set are saved.

Hyperparameter tuning was performed using grid search cross-validation (GridSearchCV) within the training set to identify the optimal parameter combinations for each classifier. To optimize model performance, ablation studies were conducted on various combinations of image filters, radiomics features and ML algorithms, ultimately identifying a good predictive model.

### 2.6. Quantification and Statistical Analysis

Statistical analysis was conducted using Python (v3.9.10). Model performance was assessed using sensitivity, specificity, balanced accuracy, and the area under the receiver operating characteristic (ROC) curve. Results are presented as the mean ± standard error (SE) with 95% confidence intervals (CIs). We conducted an exploratory power analysis [36] to assess the feasibility of the available test set sample size (infective REBLs: n = 25; neoplastic REBLs: n = 32). Assuming an anticipated effect size (Cohen’s d ≈ 0.95) [37] and a significance level of α = 0.05, the estimated power to reject the null hypothesis of no discrimination (i.e., AUC = 0.5) with this sample size was approximately 0.97 (G*Power Version 3.1.9) [38]. This indicates that the test set sample size is expected to provide substantial power to detect performance superior to chance.

## 3. Results

### 3.1. Clinical and MRI Data

A total of 149 patients who fulfilled the study inclusion criteria were included in this study. T1+C MRI brain images of 92 (infective REBLs, n = 26; neoplastic REBLs, n = 66) and 57 (infective REBLs, n = 25; neoplastic REBLs, n = 32) patients were extracted in two phases to make the datasets for model training and validation, and external holdout testing, respectively. Among the 92 patients in the training and validation set, a total of 402 REBLs were identified on the coronal plane. Among the 57 patients in the test set, a total of 454 REBLs were identified on the coronal plane. Details of data classification and splitting, and data augmentation, are shown in Table 2.

#### 3.1.1. Phase 1: Training and Validation (Model Development)—n = 92

Of the 26 patients with infective REBLs, 11 had pyogenic brain abscesses, eight tuberculosis, three *Nocardia* brain abscesses, two toxoplasmosis, one cryptococcosis and one aspergillosis (Table 3). This training dataset was augmented by including both coronal and axial T1+C images from the same patient to add anatomical diversity and data points for model training. The diagnostic criteria for “definite” and “probable” were fulfilled in 14 and 12 patients, respectively, and their respective methods of diagnosis detailed in Supplementary Table S2. Of the 66 patients with neoplastic REBLs, there were 51 with brain metastases from various primary malignancies, and 15 with gliomas (Table 3). Only coronal 3D T1+C scans were used for model training. The diagnostic criteria for “definite” and “probable” were fulfilled in 38 and 28 patients, respectively, and their respective methods of diagnosis detailed in Supplementary Table S3.

#### 3.1.2. Phase 2: External Holdout Testing—n = 57

Of the 25 and 32 patients with infective and neoplastic REBLs (Table 3), 13 and 13 fulfilled the diagnostic criteria for “definite”, while 12 and 19 fulfilled the diagnostic criteria for “probable”, respectively. Their respective methods of diagnosis are detailed in Supplementary Tables S4 and S5.

### 3.2. Radiomic Feature Extraction and Selection

A total of 1197 features were extracted from the annotated regions of 3D T1+C MR images, comprising 14 shape (3D), 234 histogram, and 286 GLCM, 208 GLRLM, 208 GLSZM, 182 GLDM and 65 NGTDM textural-based features. These features were derived from three filter groups: (a) the original image—105 features (3D shape, histogram, textural), (b) LoG filters in multiple scales—364 features (histogram and textural), and (c) Coiflets-type wavelet decomposition with combination of high-and-low pass function across 3D planes—728 features (histogram, textural). Experiments were conducted using features generated from each image filter group alone and in combination, generating a total of seven feature groupings: (i) original, (ii) LoG, (iii) wavelet, (iv) original + LoG, (v) original + wavelet, (vi) LoG + wavelet, and (vii) original + LoG + wavelet. Feature selection was performed based on MI scores. Only features which scored >0.12 were retained.

### 3.3. Model Classification Performance Evaluation

Nine ML classifiers (LR, QDA, KNN, DT, RF, SVM, AdaBoost, XGBoost, MLP) were applied to the seven feature groups to identify the best model–feature combination based on performance metrics obtained from 5-fold cross-validation, viz: (i) area under the ROC curve, (ii) sensitivity, (iii) specificity, and (iv) balanced accuracy. Results from these model–feature combinations are shown in Table 4.

**Figure 3:**
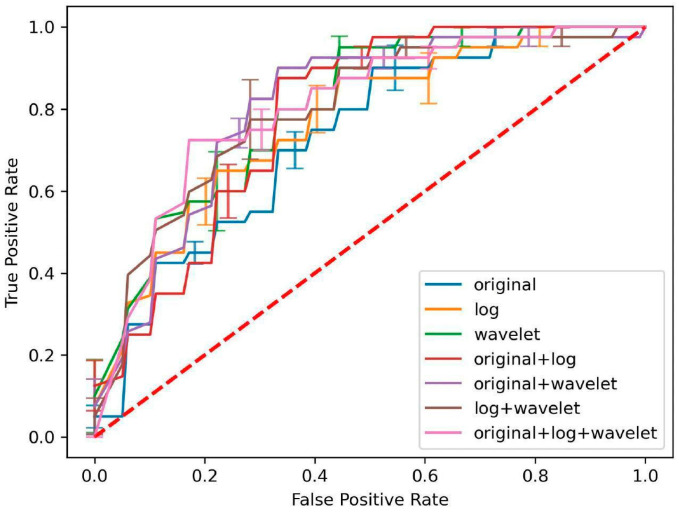
ROC curves for Multi-layer Perceptron, the best-performing classification model, in predicting infective from neoplastic ring-enhancing brain lesions on T1+C scans in the cross-validation cohort (mean ± SE, CI = 0.95), for each feature group that filtered for radiomics features with mutual information scores > 0.12.

Across all feature groups, MLP consistently demonstrated superior performance in the cross-validation cohort, with the mean area under the curve (AUC) exceeding 0.80 in several feature combination groups, namely, Original + LoG + Wavelet (0.80 ± 0.02), LoG + Wavelet (0.80 ± 0.04), Wavelet (0.80 ± 0.05) and Original + Wavelet (0.80 ± 0.03). Figure 3 illustrates the performance of MLP on ROC analysis across the seven feature groups.

This MLP model was evaluated on the unseen external holdout test data to verify its robustness in performance. The results are detailed in Table 5. AUCs were: Wavelet (0.71), Original + Wavelet (0.72), LoG (0.76), LoG + Wavelet (0.78), Original + LoG (0.84), Original + LoG + Wavelet (0.84). These results indicate stable and sustainable model performance, showing minimal drop-off between cross-validation and external testing, thereby supporting a fair and reliable comparison. Notably, the Original + LoG feature group demonstrated the best performance on the test set, with an AUC of 0.84, sensitivity of 0.88, specificity of 0.78 and balanced accuracy of 0.83.

The corresponding confusion matrices for the MLP models across feature groups are provided in Supplementary Figure S1, and the error analysis of false-negative and false-positive predictions on unseen external test set is summarized in Supplementary Table S6. In addition, the distribution of retained features by category across different filter groups and the list of the most frequently retained features are also included in Supplementary Table S7.

### 3.4. Comparative Benchmark with Deep Learning Representation

To contextualize the radiomics results against contemporary data-driven representation learning, we evaluated end-to-end CNN baselines using ResNet-10 and ResNet-18 architectures, initialized with MedicalNet-pretrained weights [39], and fine-tuned for REBLs binary classification. Model performance was assessed under the same internal 5-fold cross-validation protocol used for the radiomics experiments, and is reported as mean ± SE across folds. All comparative metrics are summarized in Table 6.

Across folds, radiomics alone was the top-performing approach, outperforming end-to-end CNN baselines and CNN + radiomics. Combining CNN-derived representations with radiomics features did not yield consistent improvement over radiomics alone, suggesting limited benefit from hybridization under the present evaluation setting.

## 4. Discussion

Despite the modest sample size for model development, our radiomics-ML model, based solely on T1+C MRI contrast in a high-resolution 3D acquisition, demonstrated good performance across AUC, sensitivity, specificity and balanced accuracy in external testing in this exploratory study. These findings highlight the clinical value and potential of 3D T1+C imaging in distinguishing infective from neoplastic REBLs.

The strength of our study lies in its high-quality clinical dataset. Board-certified infectious disease physicians meticulously reviewed each patient’s medical records to ensure that only cases with definite or probable diagnoses based on our preset criteria were included in our datasets. The 3D T1+C MRI provided high-resolution structural lesion features for radiomics analysis and allowed for improved detection and localization of small REBLs for annotation. The manual bounding box annotation of each REBL was directly supervised by board-certified neuroradiologists, serving as ground truth labels for radiomics analysis. This rigorous process of data curation provides a strong foundation for model development.

Another strength of our study is the high proportion of immunocompromised patients and the diversity of etiologies within our datasets. Around half of the patients with infective REBLs were immunocompromised. Therefore, we were able to include diverse etiologies of infective REBLs, including various opportunistic infections, in our datasets. Similarly, our oncology service sees patients with diverse malignancies. The intentional inclusion of heterogeneous etiologies in our datasets for model development reflects the epidemiology and the rich radio-pathological case mix of CNS infections and neoplasms at our center. As the patient who presents with REBL is often undifferentiated, and differential diagnoses typically extend beyond two etiologies, models that distinguish abscess from a specific neoplasm may possess limited potential for deployment in a real-world clinico-radiology workflow [29,30,32], compared to our model which distinguishes infective REBLs from neoplastic REBLs. Accordingly, the clinical dilemma of infection versus neoplasm faced by the on-duty radiologist could be better resolved by our model. While our model does not yield a specific diagnosis, rapid and accurate classification would assist clinicians in selecting appropriate management strategies—those assessed to have infection are aggressively managed by expedited abscess drainage and antibiotics, while those assessed to have neoplasm are systemically evaluated, including assessment for an extracranial primary tumor that may be safer to biopsy. Consequently, unnecessary brain biopsy, and associated neurological complications may potentially be avoided.

Deep learning architectures, especially CNNs offer automatic feature learning. However, they typically require substantially larger datasets to achieve stable convergence and generalizable performance [34,40]. Given our limited sample size, a radiomics-based framework was adopted to ensure interpretability and robustness within the constraints of this exploratory study. To contextualize our radiomics findings against modern representation learning, we explored end-to-end CNN baselines. Compared with radiomics alone, both CNN baselines demonstrated poorer performance. This was similarly observed in other studies [41,42], where deep representations did not consistently outperform hand-crafted radiomics on independent testing since representation learning generally benefits from larger and more diverse datasets to achieve stable generalization. Increasing network depth from ResNet-10 to ResNet-18 did not yield improved discrimination in this cohort, suggesting that additional model capacity did not translate into better generalization under the present data constraints. Additionally, we evaluated hybrid models that combined radiomics features with CNN-extracted representations to assess potential complementarity between engineered and learned features. Similarly, these hybrid configurations did not demonstrate improvement over radiomics alone. Unlike Bo et al. [29], deep learning-based radiomics features did not result in a better performance than radiomics alone despite our superior lesion count, as their setting involved a narrower binary task with more modality inputs than the present study.

We included a wide range of ML classifiers in our exploratory evaluation. MLP demonstrated superior performance in distinguishing infective from neoplastic REBLs due to several key factors. The model architecture in MLP allows it to effectively capture complex, non-linear relationships within the data that are problematic with simpler models like KNN and DT, as well as with classifiers constrained by linear or quadratic decision functions (LR and QDA) or margin-based separation (SVM). The deep architecture of the MLP is also efficacious in leveraging feature combinations of original + LoG + wavelet, allowing it to better understand the diverse characteristics of the data compared to ensemble methods like Random Forest, AdaBoost and XGBoost. Additionally, the iterative training process of MLP facilitates hyperparameter optimization, enhancing the model’s adaptability and performance for the specific dataset. Among the three radiomic feature categories, texture-based features were the most influential in distinguishing infective from neoplastic REBLs (Supplementary Table S7). These features quantify intra-lesional heterogeneity and edge sharpness, which are radiologically useful in differentiating neoplastic lesions from abscesses. Shape and intensity-based features contributed complementary morphological and statistical information that also enhanced overall model robustness.

The main limitation of our study is the small sample size of patients with infective REBLs, which we mitigated with data augmentation. While the meticulous review by infectious diseases physicians allowed for the inclusion of patients whose microbiological diagnoses were made from blood or extracranial tissue, reflecting current diagnostic approaches, factors such as our stringent patient inclusion criteria, the lower incidence of infective REBLs compared to neoplastic REBLs, the high mortality of CNS infections (often prior to achieving microbiological diagnosis), and the exclusion of patients without a contrast-enhanced MRI resulted in the relatively small cohort. This challenge was similarly encountered by other authors [29,30,32] in slightly different CNS infection versus neoplasm use cases (Supplementary Table S1). The sample-size-related limitation highlights the need for further evaluation of overfitting and feature selection stability in future work. To mitigate the limitation, in contrast to other models [29,30,32] in which only one lesion per patient was manually segmented, every lesion of each patient was manually segmented, providing a superior lesion count that far outnumbers that of these other studies [29,30,32] and optimizing the data points for our model training.

The majority of the neoplastic lesions that were wrongly classified by our model were metastases (Supplementary Table S6) while fewer primary brain tumors (astrocytoma, glioblastoma) were wrongly classified. This correlates well with clinical practice, as cystic metastases resemble abscesses on T1+C, while primary brain tumors tend to have thicker walls and different enhancement patterns than metastases. In particular, small lesion size and smooth thin walls with little wall thickening were features most associated with wrong classification among patients with neoplastic REBLs. Future study with inclusion of additional MR sequences, especially DWI which is clinically useful in abscess vs. tumor differentiation, could improve our model performance.

During the review process for the training/validation set, we had purposefully excluded cases in which no pathogen or neoplasm was identified as well as those whose clinical/radiological response to treatment was suboptimal as these patients may have mixed lesions, i.e., both infection and neoplasm in the same patient. While this ensured that model training was based on a dataset with high diagnostic certainty, there may be selection bias as certain patients, such as those with mixed lesions and those who died before a microbiological/pathological diagnosis can be determined, were excluded from our study. Our model may not perform well in these patients, but would surface uncertainty in classification accordingly, triggering the need for greater attention by clinicians. Importantly, while our radiomics-ML may aid with diagnosis, it should not replace clinical judgement, and the provisional diagnosis should always be revisited and revised when clinical response is not as expected.

While our model, trained on a dataset encompassing a broad range of etiologies, offers greater potential for deployment in a real-world clinico-radiology workflow compared to models that differentiate between two specific pathologies, its performance may be impacted by the heterogeneity of etiologies within the broad diagnostic categories of infective and neoplastic REBLs. This heterogeneity may obscure clinically relevant biological, MRI, and radiomic differences between etiologies within each category, potentially limiting diagnostic precision.

The model was trained on a single-center dataset from the largest tertiary referral hospital in our country, affording a rich case mix of CNS infections and neoplasms. External validation on a dataset from a second tertiary center showed comparable performance, supporting generalizability. Further multicenter validation and the application of harmonization techniques such as ComBat to account for batch effects across institutions with different MRI protocols can further improve generalizability. The complementary value of spatial information as related to known spatial predilections of different pathologies was also not assessed in our study. While N4 bias-field correction was not applied, several factors may limit its impact on our findings: (1) radiomic features were extracted from localized ROIs rather than whole-brain regions, minimizing intra-ROI bias-field variation; and (2) all scans were acquired on scanners from the same vendor with built-in prescan normalization.

In this exploratory study, our radiomics model, based solely on a 3D T1+C dataset, demonstrated potential in distinguishing infective from neoplastic REBLs. This finding emphasizes the value of high-resolution 3D T1+C datasets in clinical radiology for quantitative analytics downstream beyond radiological reading and surgical planning. In addition, a time-efficient bounding box approach to lesion localization enabled inclusion of multiple REBLs for model development, and this could be further scoped for automated contour-based REBL segmentation in the next phase. Incorporating additional MR sequences—particularly DWI, which is clinically useful in abscess vs. tumor differentiation—along with clinical data within a multimodal MRI radiomics-ML framework is likely to further enhance discriminative performance and clinical applicability, and certainly warrants further study. Recently, multimodal radiomics models that combine multiple MRI sequences and different imaging modalities (e.g., perfusion MRI) have shown potential in cancer diagnosis and prognostication [43,44,45,46]. These necessitate accurate image co-registration across different MRI sequences, which brings attendant challenges, especially in restless patients who might have moved between sequence acquisitions. A multimodal radiomics model could achieve a better performance, but would also face challenges such as large computing demands and limited generalizability in the absence of protocol harmonization across institutions.

## 5. Conclusions and Future Work

Rapid and accurate classification of REBLs is crucial to guide subsequent diagnostic evaluation and treatment, which are vastly different between infection or neoplasm. Our exploratory radiomics-ML model, built upon high-quality 3D imaging data and expert-curated clinical labels, achieved stable results across internal validation and external testing cohorts. Among nine ML algorithms evaluated, the MLP model consistently demonstrated the best performance. In a comparative benchmark, end-to-end CNN baselines and hybrid radiomics-CNN models did not show consistent improvement over radiomics alone under the present evaluation setting. Our results highlight the clinical value and potential of 3D T1+C radiomics in distinguishing infective from neoplastic REBLs.

Future investigation with larger, balanced multicenter datasets and integration of clinical and multimodal imaging data (additional MRI sequences, such as DWI will enhance diagnostic accuracy and generalizability. Incorporating explainable AI techniques such as SHAP or LIME could further improve model transparency and strengthen clinician confidence by identifying the radiomic features most influential to classification. A multimodal, interpretable radiomics-ML framework, that can rapidly and accurately classify REBLs into infection or neoplasm, would be valuable as a decision-support tool in the 24/7 clinico-radiology workflow. This capability could facilitate timely and appropriate diagnostic evaluation and treatment, ultimately contributing to improved patient outcomes.

## Figures and Tables

**Table 1:** MRI acquisition parameters.

Scanner Model	Siemens Magnetom Avanto		Siemens Magnetom Skyra	
Field strength	1.5T		3T	
Sequence	2D axial SE	3D coronal GRE	2D axial SE	3D coronal GRE
TR (ms)	400–490	1100–1300	500–530	1800–1900
TE (ms)	11–17	3.58–4.73	8.4–9.2	2.26
TI (ms)	N/A	600	N/A	900–950
FA (°)	90	15	70–80	8–9
Voxel size (mm^3^)	0.65 × 0.65 × 5	1 × 1 × 1	0.86 × 0.86 × 5	1 × 1 × 1
Acq matrix	320 × 202	256 × 208	256 × 186	256 × 208
Number of slices	22–24	208–224	30	224–240

N/A: No TI for SE sequence.

**Table 2:** Dataset classification and split for model development (training, validation) and external holdout testing.

	Training (n = 66)		Validation (n = 26)	Testing (n = 57)
	Coronal T1+C	Axial T1+C		
Infection	18	18	8	25
Neoplasm	48	NA	18	32
Total (T1+C datasets)	84		26	57

n = number of unique patients. NA = denotes no need for data augmentation in the neoplasm group.

**Table 3:** Underlying etiologies of infective ring-enhancing brain lesions confirmed on clinico-microbiological and/or histopathological data.

Etiologies	Number of Patients	
	Training and Validation	Testing
Infective REBLs		
Pyogenic brain abscess	11	10
Tuberculosis	8	8
*Nocardia* brain abscess	3	1
Toxoplasmosis	2	4
Cryptococcosis	1	1
Aspergillosis	1	1
Subtotal	26	25
Neoplastic REBLs		
Glioma	15	8
Breast cancer	13	7
Lung cancer	13	8
Colorectal cancer	8	3
Lymphoma	6	2
Gynecological cancer	3	0
Renal cancer	2	1
Sarcoma	2	0
Unknown primary	2	1
Melanoma	1	1
Pancreatic cancer	1	0
Germ cell tumor	0	1
Subtotal	66	32
Total	92	57

**Table 4:** Summary of cross-validation performance of nine machine learning classifiers across different filter groups (mean ± SE, 95% CI), values > 0.75 in bold.

Filter Group	LR	QDA	KNN	DT	RF	SVM	AdaBoost	XGBoost	MLP
AUC (mean ± SE)									
Original	0.60 ± 0.05	0.58 ± 0.05	0.55 ± 0.04	0.51 ± 0.07	0.65 ± 0.05	0.59 ± 0.06	0.65 ± 0.05	0.54 ± 0.07	0.73 ± 0.03
LoG	0.63 ± 0.07	0.59 ± 0.07	0.50 ± 0.04	0.62 ± 0.04	0.71 ± 0.06	0.67 ± 0.06	0.71 ± 0.06	0.67 ± 0.10	**0.77 ± 0.05**
Wavelet	0.59 ± 0.05	0.54 ± 0.04	0.56 ± 0.05	0.59 ± 0.08	0.64 ± 0.10	0.67 ± 0.09	0.64 ± 0.10	0.56 ± 0.09	**0.80 ± 0.05**
Original + LoG	0.69 ± 0.05	0.67 ± 0.04	0.64 ± 0.10	0.64 ± 0.07	0.69 ± 0.06	**0.78 ± 0.06**	0.69 ± 0.06	0.65 ± 0.10	**0.78 ± 0.02**
Original + Wavelet	0.60 ± 0.05	0.52 ± 0.04	0.54 ± 0.09	0.57 ± 0.08	0.65 ± 0.07	0.68 ± 0.05	0.65 ± 0.07	0.53 ± 0.09	**0.80 ± 0.03**
LoG + Wavelet	0.61 ± 0.07	0.53 ± 0.01	0.58 ± 0.05	0.61 ± 0.10	0.67 ± 0.08	0.71 ± 0.07	0.67 ± 0.08	0.61 ± 0.08	**0.80 ± 0.04**
Original + LoG + Wavelet	0.66 ± 0.07	0.62 ± 0.04	0.53 ± 0.07	0.59 ± 0.09	0.69 ± 0.09	**0.79 ± 0.06**	0.69 ± 0.09	0.61 ± 0.09	**0.80 ± 0.02**
Sensitivity (mean ± SE)									
Original	**0.88 ± 0.10**	0.65 ± 0.15	0.55 ± 0.16	0.30 ± 0.17	**0.88 ± 0.06**	0.59 ± 0.06	**0.88 ± 0.06**	0.58 ± 0.13	**0.90 ± 0.07**
LoG	0.60 ± 0.11	**0.95 ± 0.04**	0.63 ± 0.24	0.55 ± 0.04	**0.80 ± 0.10**	0.67 ± 0.06	**0.80 ± 0.10**	0.70 ± 0.17	**0.83 ± 0.10**
Wavelet	0.37 ± 0.14	0.32 ± 0.10	0.30 ± 0.07	0.43 ± 0.23	**0.78 ± 0.10**	0.67 ± 0.09	**0.78 ± 0.10**	0.73 ± 0.13	**0.90 ± 0.08**
Original + LoG	0.67 ± 0.12	**0.88 ± 0.05**	0.58 ± 0.21	0.53 ± 0.10	0.60 ± 0.12	**0.78 ± 0.06**	0.60 ± 0.12	0.60 ± 0.21	**0.95 ± 0.03**
Original + Wavelet	0.62 ± 0.13	0.23 ± 0.08	0.58 ± 0.21	0.38 ± 0.20	**0.88 ± 0.06**	0.68 ± 0.05	**0.88 ± 0.06**	**0.80 ± 0.07**	**0.90 ± 0.06**
LoG + Wavelet	0.40 ± 0.11	0.23 ± 0.04	0.33 ± 0.07	0.45 ± 0.17	**0.75 ± 0.11**	0.71 ± 0.07	**0.75 ± 0.11**	0.68 ± 0.19	**0.85 ± 0.08**
Original + LoG + Wavelet	0.65 ± 0.10	**0.87 ± 0.03**	0.45 ± 0.21	0.40 ± 0.15	**0.83 ± 0.04**	**0.79 ± 0.06**	**0.83 ± 0.04**	0.53 ± 0.22	**0.83 ± 0.09**
Specificity (mean ± SE)									
Original	0.26 ± 0.08	0.38 ± 0.13	0.63 ± 0.16	**0.79 ± 0.12**	0.51 ± 0.08	0.30 ± 0.06	0.51 ± 0.08	0.70 ± 0.13	0.58 ± 0.05
LoG	0.50 ± 0.10	0.22 ± 0.03	0.51 ± 0.26	0.69 ± 0.12	0.64 ± 0.12	0.46 ± 0.05	0.64 ± 0.12	0.71 ± 0.09	0.68 ± 0.08
Wavelet	0.70 ± 0.10	**0.78 ± 0.04**	**0.90 ± 0.06**	**0.79 ± 0.12**	0.57 ± 0.15	0.66 ± 0.16	0.57 ± 0.15	0.54 ± 0.16	0.68 ± 0.13
Original + LoG	0.62 ± 0.07	0.26 ± 0.10	0.74 ± 0.10	**0.76 ± 0.13**	**0.78 ± 0.13**	0.62 ± 0.08	**0.78 ± 0.13**	**0.80 ± 0.10**	0.61 ± 0.05
Original + Wavelet	0.68 ± 0.06	**0.84 ± 0.03**	0.64 ± 0.21	**0.81 ± 0.10**	0.50 ± 0.12	**0.80 ± 0.13**	0.50 ± 0.12	0.42 ± 0.11	0.72 ± 0.04
LoG + Wavelet	**0.86 ± 0.06**	**0.82 ± 0.03**	**0.90 ± 0.07**	**0.82 ± 0.08**	0.63 ± 0.14	0.70 ± 0.09	0.63 ± 0.14	0.66 ± 0.17	0.74 ± 0.09
Original + LoG + Wavelet	0.62 ± 0.06	0.20 ± 0.05	0.74 ± 0.17	**0.84 ± 0.07**	0.60 ± 0.08	0.68 ± 0.07	0.60 ± 0.08	**0.87 ± 0.08**	**0.77 ± 0.08**
Balanced Accuracy (mean ± SE)									
Original	0.57 ± 0.05	0.52 ± 0.05	0.59 ± 0.02	0.54 ± 0.04	0.69 ± 0.04	0.51 ± 0.06	0.69 ± 0.04	0.64 ± 0.04	0.74 ± 0.02
LoG	0.55 ± 0.05	0.61 ± 0.03	0.57 ± 0.03	0.62 ± 0.04	0.72 ± 0.03	0.62 ± 0.05	0.72 ± 0.03	0.71 ± 0.07	**0.75 ± 0.03**
Wavelet	0.54 ± 0.04	0.55 ± 0.05	0.60 ± 0.04	0.61 ± 0.07	0.67 ± 0.06	0.65 ± 0.06	0.67 ± 0.06	0.63 ± 0.06	**0.79 ± 0.04**
Original + LoG	0.65 ± 0.03	0.57 ± 0.02	0.66 ± 0.06	0.64 ± 0.07	0.69 ± 0.03	0.70 ± 0.03	0.69 ± 0.03	0.70 ± 0.07	**0.78 ± 0.02**
Original + Wavelet	0.65 ± 0.03	0.53 ± 0.01	0.61 ± 0.05	0.59 ± 0.06	0.69 ± 0.04	0.69 ± 0.03	0.69 ± 0.04	0.61 ± 0.06	**0.81 ± 0.03**
LoG + Wavelet	0.63 ± 0.03	0.52 ± 0.01	0.61 ± 0.05	0.64 ± 0.06	0.69 ± 0.05	0.68 ± 0.02	0.69 ± 0.05	0.67 ± 0.04	**0.80 ± 0.03**
Original + LoG + Wavelet	0.63 ± 0.03	0.55 ± 0.02	0.60 ± 0.04	0.62 ± 0.05	0.71 ± 0.05	0.72 ± 0.03	0.71 ± 0.05	0.70 ± 0.07	**0.80 ± 0.02**

**Table 5:** Multi-layer Perceptron (MLP) model performance on unseen external holdout testing cohort with different feature combinations, values > 0.75 in bold.

Group	AUC	Sensitivity	Specificity	Balanced Accuracy
Original	0.66	**0.80**	0.50	0.65
LoG	**0.76**	0.72	**0.84**	**0.78**
Wavelet	0.71	0.64	**0.81**	0.73
Original + LoG	**0.84**	**0.88**	**0.78**	**0.83**
Original + Wavelet	0.72	**0.80**	0.66	0.73
LoG + Wavelet	**0.78**	**0.80**	0.66	0.73
Original + LoG + Wavelet	**0.84**	**0.84**	**0.75**	**0.80**

**Table 6:** Comparative benchmark between radiomics (Original + LoG + Wavelet) and end-to-end CNN representations under 5-fold cross-validation (mean ± SE).

Model	AUC	Sensitivity	Specificity	Balanced Accuracy
ResNet-10	0.76 ± 0.04	0.63 ± 0.09	0.89 ± 0.04	0.76 ± 0.04
ResNet-18	0.75 ± 0.03	0.83 ± 0.07	0.66 ± 0.11	0.74 ± 0.03
ResNet-10 + Radiomics	0.80 ± 0.03	0.78 ± 0.04	0.74 ± 0.03	0.80 ± 0.03
ResNet-18 + Radiomics	0.77 ± 0.03	0.83 ± 0.03	0.77 ± 0.03	0.80 ± 0.03
Radiomics	0.80± 0.02	0.83 ± 0.09	0.77 ± 0.08	0.80 ± 0.02

## Data Availability

This paper utilizes publicly available code in development of the machine learning model. Iteration of the model available on request from the lead contact.

## Supplementary Materials

The following supporting information can be downloaded at: https://www.mdpi.com/article/10.3390/diagnostics16060926/s1, Supplementary Table S1. Comparison of models that distinguish brain abscess from neoplasm; Supplementary Data S1. Search terms indicating ring-enhancing brain lesions on radiological reports; Supplementary Data S2. Annotation protocol; Supplementary Table S2. Microbiological diagnosis of cases of infective REBLs in training/validation set and the respective method of diagnosis; Supplementary Table S3. Histological diagnosis of cases of neoplastic REBLs in training/validation set and the respective method of diagnosis; Supplementary Table S4. Diagnoses of the cases of infective REBLs in the external holdout test set and the respective method of diagnosis; Supplementary Table S5. Diagnoses of the cases of neoplastic REBLs in the external holdout test set and the respective method of diagnosis; Supplementary Figure S1. Confusion matrices of the MLP-based radiomics models across feature groups in the cross-validation and independent test set; Supplementary Table S6. Error analysis of false-negative and false-positive cases for the MLP-based radiomics models on the independent test set across radiomic feature groups: Original (O), Laplacian of Gaussian (L), Wavelet (W), and their combinations; Supplementary Table S7. Distribution of retained radiomic features by category (shape, intensity and textural) across different filter groups.

## Abbreviations

The following abbreviations are used in this manuscript:

2D	Two-dimensional
3D	Three-dimensional
AUC	Area under the curve
CI	Confidence interval
CNS	Central nervous system
CSF	Cerebrospinal fluid
CT	Computed tomography
DT	Decision Tree
DWI	Diffusion-weighted imaging
FA	Flip angle
FLAIR	Fluid-attenuated inversion recovery
GLCM	Grey-level co-occurrence matrix
GLDM	Grey-level dependence matrix
GLSZM	Grey-level size zone matrix
HIV	Human immunodeficiency virus
KNN	K-Nearest Neighbors
LoG	Laplacian of Gaussian
LR	Linear Regression
MI	Mutual information
ML	Machine learning
MLP	Multi-layer Perceptron
MRI	Magnetic resonance imaging
NGTDM	Neighboring grey tone difference matrix
QDA	Quadratic Discriminant Analysis
RF	Random Forest
REBL	Ring-enhancing brain lesion
ROC	Receiver operating characteristic
SVM	Support Vector Machine
T1+C	Contrast-enhanced T1-weighted
TE	Echo time
TI	Inversion time
TR	Repetition time
