# Supplementary material for: Can 3D T1 Post-Contrast MRI in A Radiomics-Machine Learning Model Distinguish Infective from Neoplastic Ring-Enhancing Brain Lesions? An Exploratory Study
Source: Diagnostics (Basel). 2026 Mar 20;16(6):926. doi: 10.3390/diagnostics16060926 (PMC13024827; doi:10.3390/diagnostics16060926)
Supplement: Supplementary file 1 [file diagnostics-16-00926-s001.zip › diagnostics-4135574-supplementary.pdf]

Supplementary Table S1. Comparison of models that distinguish brain abscess from neoplasm

**Authors**	Bo, L.; et al.[29]	Xiao, D.; et al.[30]	Cui, L.; et al.[32]	Sng.; et al.
**Published year**	2021	2021	2024	2026
**Patient Dataset**	86 patients with cystic glioma, 102 patients with brain abscess	86 patients with necrotic glioblastoma, 32 patients with brain abscess	98 patients with cerebral cystic metastases, 88 patients with brain abscesses	98 patients with neoplastic ring-enhancing brain lesions, 51 patients with infective ring-enhancing brain lesions
**Lesion Count**	188	118	186	849
**Imaging Dataset**	T1WI/T2WI (2D 5 mm thick slices/1 mm gap), largest cross-sectional area of one lesion manually segmented per patient	T1+C for REBL, T2-FLAIR for perilesional edema (2D images, slice thickness 4–6 mm), VOI of one abscess/tumor manually segmented per patient	DWI (5-6 mm slices/1 mm gap, largest lesion manually segmented on T1+C	3D T1+C (1 mm^3 isotropic voxels), manual segmentation of all REBLs in each patient
**Machine-learning/ Deep-learning Technique**	Deep-transfer learning (VGG-19 and ResNet) and hand-crafted radiomics features (1st order, texture, shape, histogram); Pyradiomics feature extraction, Spearman rank correlation, MI, LASSO and RFE	Clinical (neutrophil count), peritumoral edema/ tumor volume ratio and Pyradiomics feature extraction (1st order, shape, texture, LoG and wavelet filters); LASSO, RF ranking, PCA	Radiomics features (1st order, shape, gray-level, wavelet filters) from core, wall, combined; machine learning (Tree-based Pipeline Optimization Tool), compared against a clinical model based on clinical and imaging characteristics	Radiomics features (shape, histogram, texture, LoG and wavelet filters); machine learning classification models

**Model Performance**	AUC 0.86 (training)<br>AUC 0.85 (testing)	AUC 0.993 (training)<br>AUC 0.907 (testing)	AUC 1.00 (training)<br>AUC 1.00 (testing, external)	AUC 0.80 (training)<br>AUC 0.84 (testing, external)
**Limitations**	Nature of abscesses not provided; thick slice, only used slice containing largest cross- sectional area of lesion. Differentiates brain abscess from a specific neoplastic etiology; single centre (no external test).	Small abscess patient cohort, bacterial abscess vs necrotic glioblastoma only use cases; thick MRI slices; single centre (no external test).	Strict inclusion criteria: uniform lesion appearance within each category (metastases were completely cystoid with enhancement wall and cystic fluid core; all cases of brain abscesses were in the capsule stage.) Included abscesses without pathogen identified; single sequence (DWI);. thick DWI slices; differentiates brain abscess from a specific neoplastic etiology.	Single sequence (T1+C). Lack clinical features.
**Significance of findings**	T2WI based combined deep-transfer learning and hand-crafted radiomics features can distinguish brain abscesses from cystic glioma.	Combined whole tumor radiomics and peritumoral edema/ tumor volume ratio model provided best classification performance.	DWI-based radiomics shows promise in distinguishing cerebral cystic metastases from abscesses.	3D T1+C shows good potential for inclusion in a multi-modal MRI radiomics-machine learning model to distinguish infective from neoplastic REBLs.

T1+C = T1 post-contrast; REBL = ring-enhancing brain lesions; FLAIR = fluid-attenuated inversion recovery; VOI = volume of interest; WI = weighted image; DWI = Diffusion Weighted Imaging; LoG = Laplacian of Gaussian; LASSO = Least Absolute Shrinkage and Selection Operator; RF = random forest; PCA = principal component analysis; VGG = Visual Geometry Group; MI = mutual information; RFE = recursive feature elimination; AUC = area under the curve; ROI = region of interest

Supplementary Data S1. Search terms indicating ring-enhancing brain lesions on radiological reports

1. ring; ring-enhancing; ring enhancing; ring-enhancement; ring enhancement; wall-enhancement
2. rim, rim-enhancing; rim enhancing; rim-enhancement; rim enhancement; irregular wall enhancement
3. ring; ring lesion; ring mass; ring cystic; incomplete ring enhancement
4. lobular enhancing
5. abscess
6. infective lesion; infective lesions
7. septic lesion; septic lesions
8. necrotic centre; centrally necrotic; central necrosis
9. thin-walled; thin walled cystic; thin enhancing walls
10. thick-walled; thick-walled cystic; thick enhancing walls
11. cystic; cystic nodule; cystic mass; cystic lesion
12. pus; pussy; purulent
13. contents of restricted diffusion; contents with restricted diffusion; contents of low ADC; contents with low ADC; central low ADC; heterogenous restricted diffusion
14. enhancing walls
15. round cyst; round cystic; rounded cyst; tense cystic
16. T2 hypointense walls; T2-hypointense walls; T2 hypointense wall; T2-hypointense walls
17. bilobed; multilobular; multicystic; multi-cystic; complex solid-cystic; partially solid-cystic; multi-septated; mixed cystic-solid
18. internal projections
19. satellite lesions; satellite lesion
20. haematogenous spread of infection
21. central liquefaction

Supplementary Data S2. Annotation protocol.

1. Choose our project named REBL on XNAT.
    a. Click "Browse" -> Select our project i.e. "REBL"

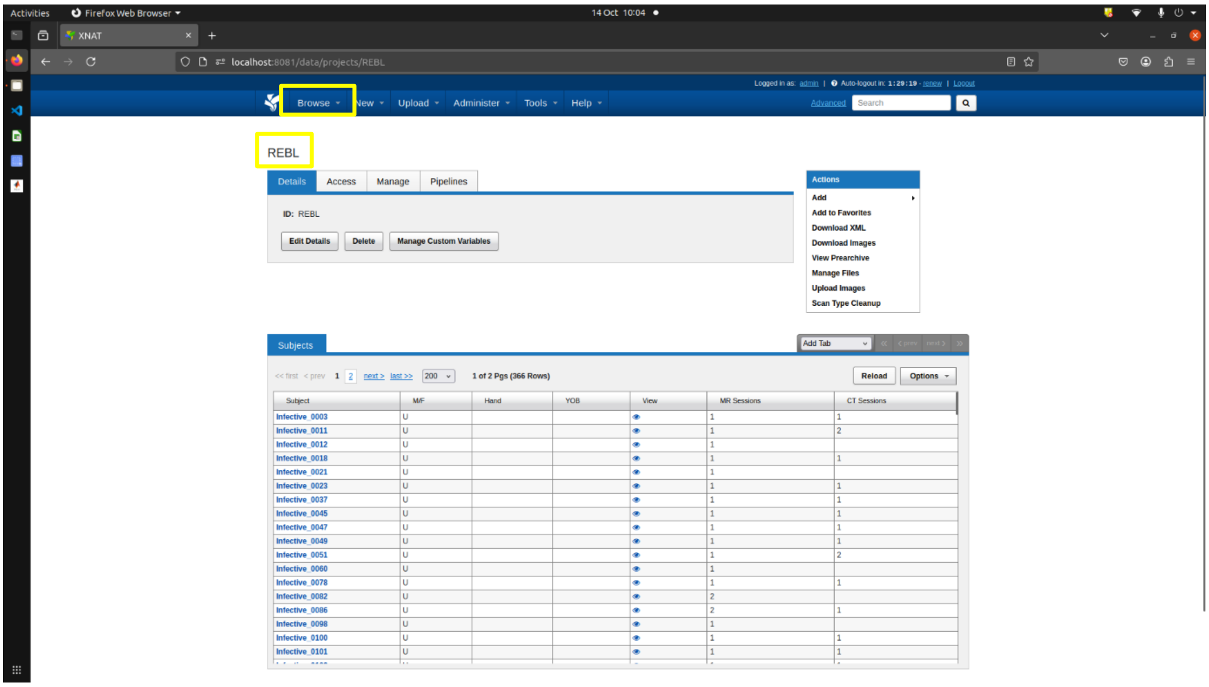

2. Select the subject to annotate.

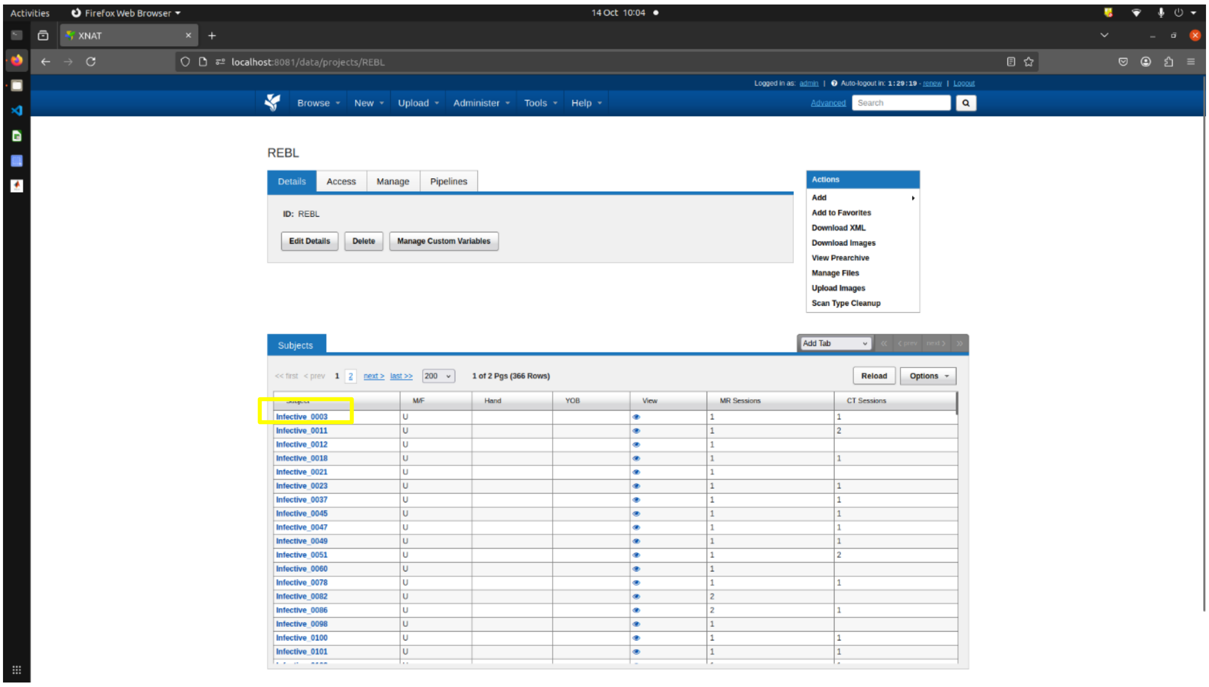

3. Select the MR Session to annotate.

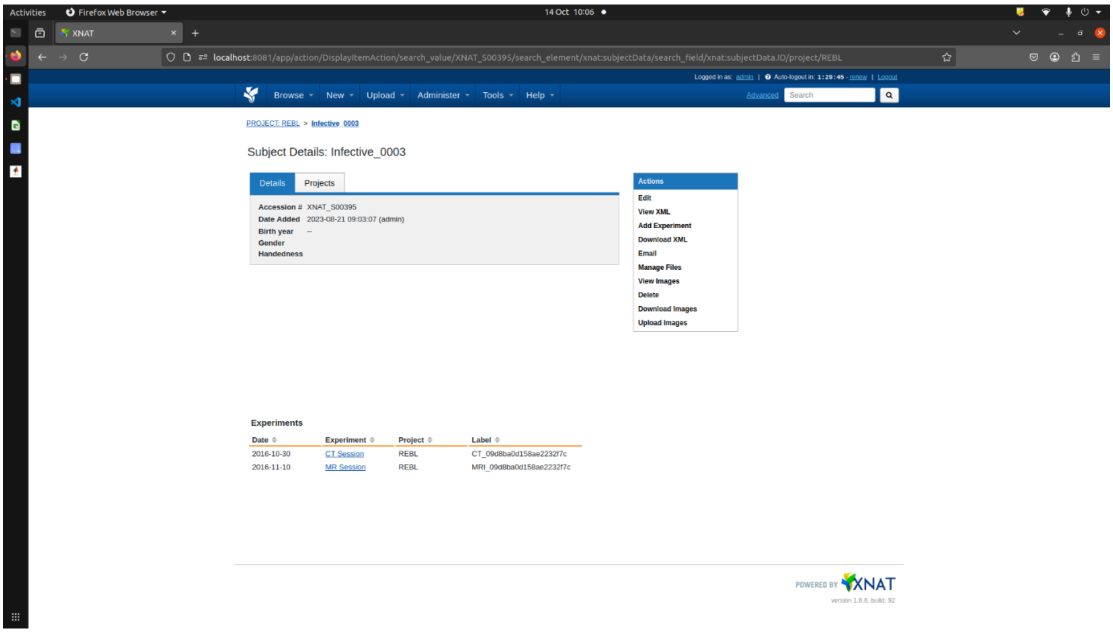

4. Click "View Images" to open the images.

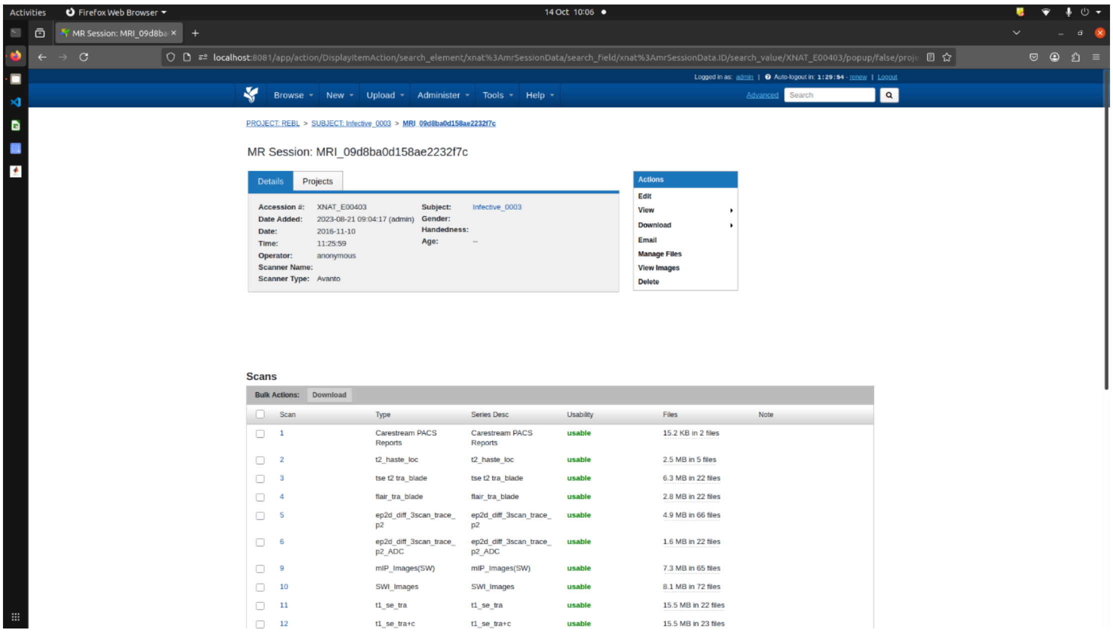

5. Select the preferred layout and open axial and coronal images of T1 with contrast.

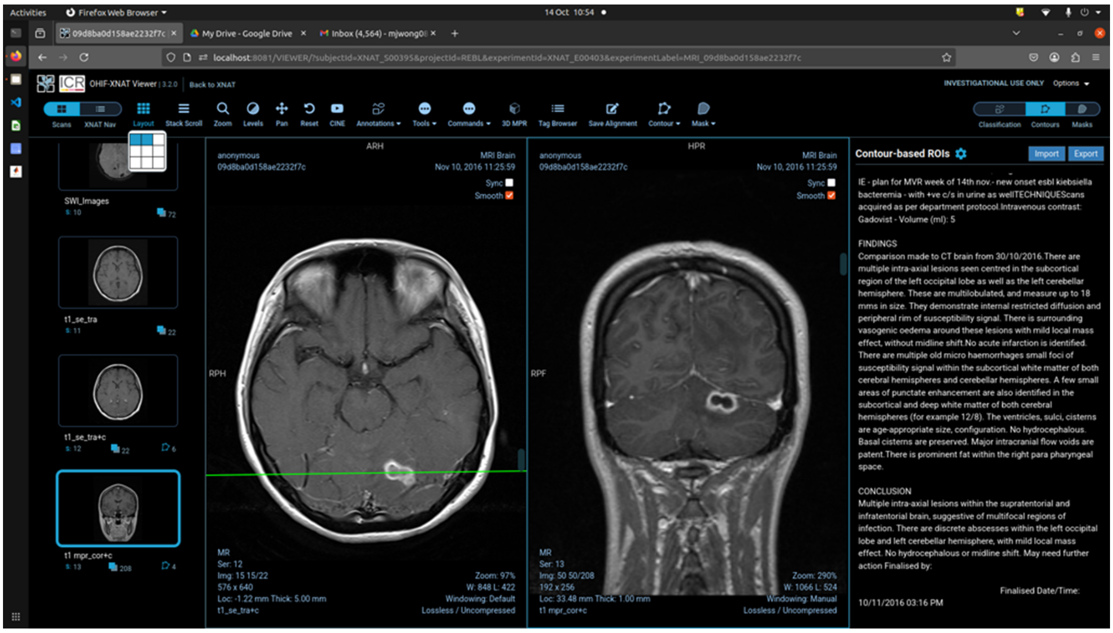

6. Select the “Contour”, “Rectangle” tool.

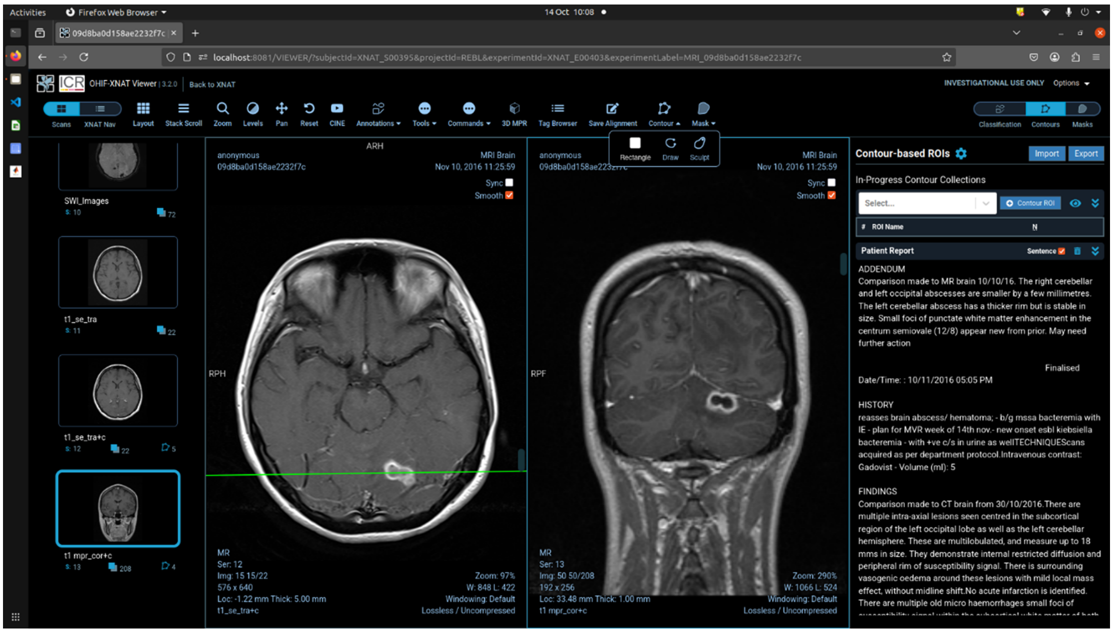

7. Name the lesion to be annotated and click “Contour ROI” to start annotation.

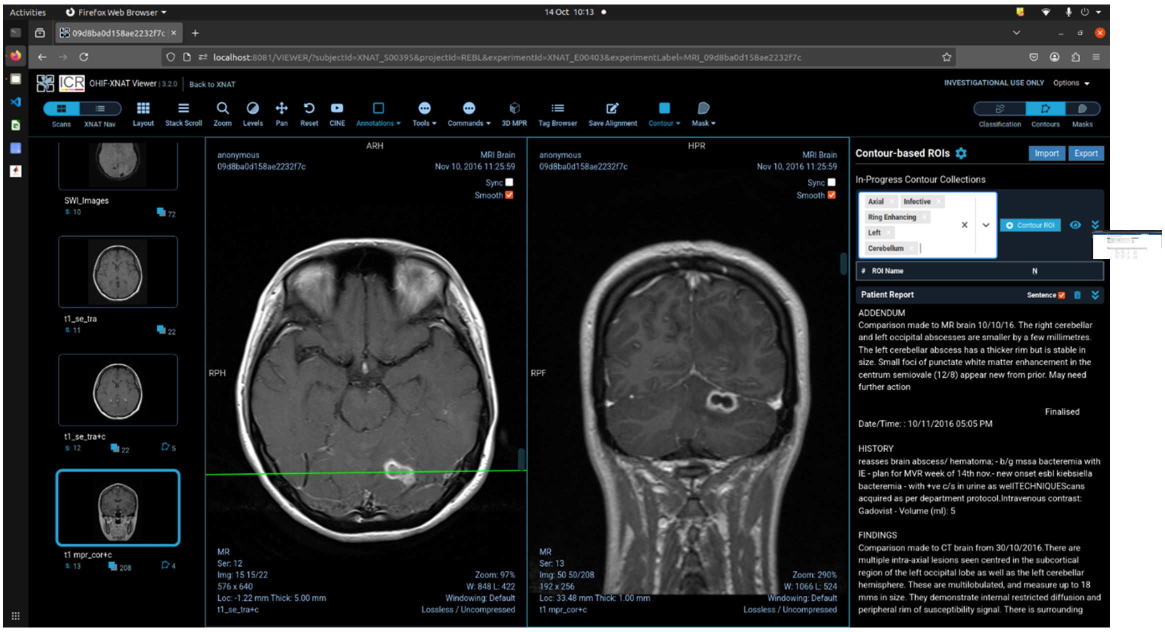

8. Select the lesion name and annotate the lesion on all slices showing the lesion.

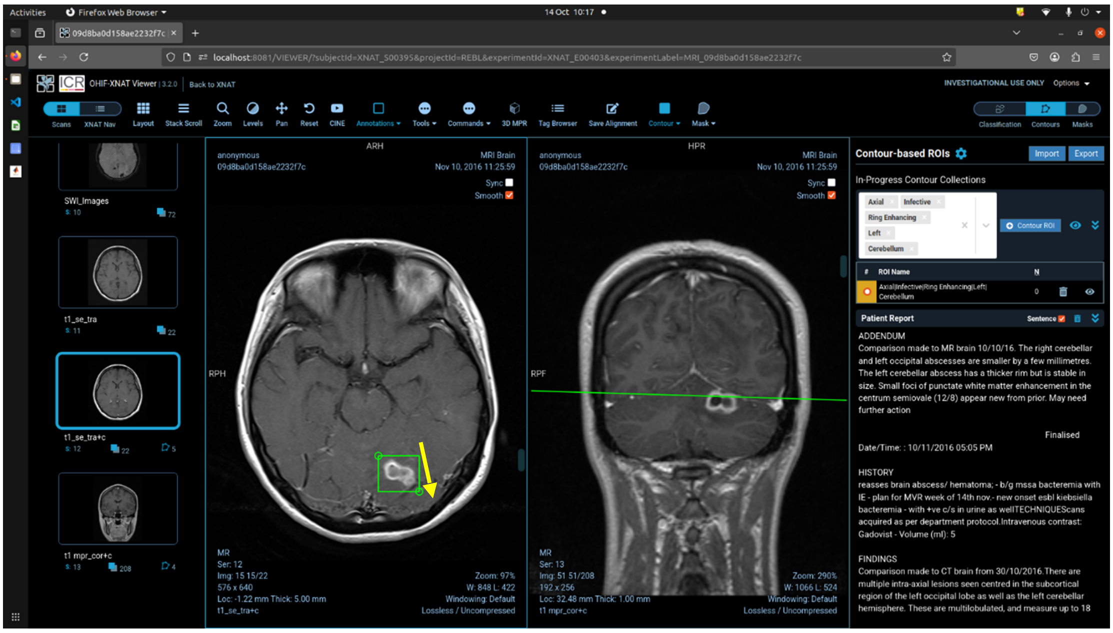

9. Repeat Steps 7 and 8 for all other lesions of the subject.

10. After finishing annotations of all lesions, export the annotations to save.

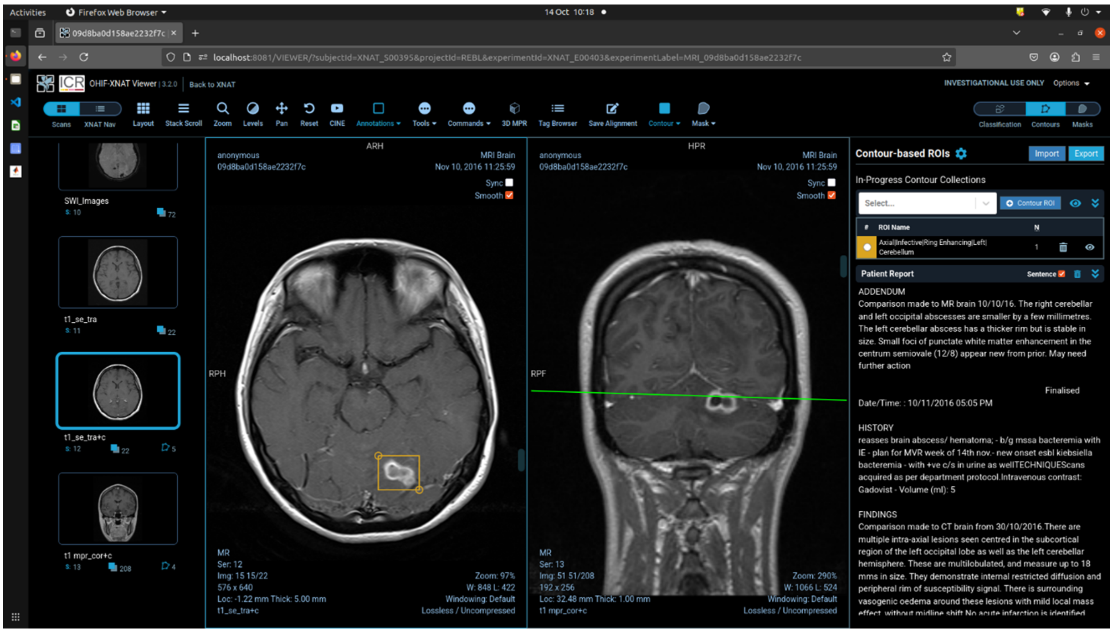

11. Name the annotation eg. Axial/Coronal_Version1.

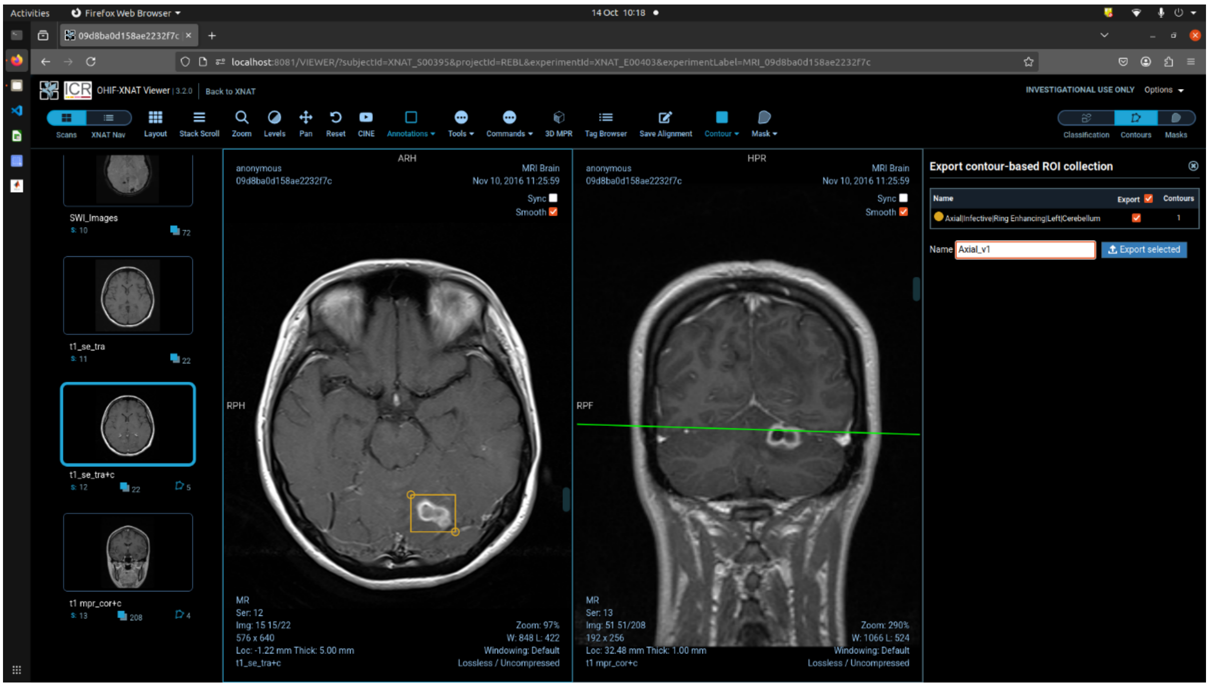

12. Repeat Steps 6-11 for Coronal T1+C.

13. To edit the exported annotations, click “Import” and select the annotation. After editing, export again by repeating Steps 10 and 11.

Supplementary Table S2. Microbiological diagnosis of cases of infective REBLs in training/validation set and the respective method of diagnosis

	Diagnosis	Diagnostic certainty	Method of diagnosis
1	*Klebsiella pneumoniae* brain abscess	Definite	Brain abscess culture
2	*Klebsiella pneumoniae* brain abscess	Definite	CSF culture
3	*Klebsiella pneumoniae* brain abscess	Definite	Brain abscess culture
4	*Klebsiella pneumoniae* brain abscess	Probable	Blood culture
5	Polymicrobial pyogenic brain abscess (*Fusobacterium*, gram positive cocci and gram positive bacilli on gram stain)	Definite	Brain abscess gram stain and 16S rRNA sequencing
6	Polymicrobial pyogenic brain abscess (*Streptococcus intermedius, Eikenella corrodens*)	Definite	Brain abscess culture
7	Polymicrobial (*Weisella viridescens, Fusobacterium*) brain abscess	Definite	Blood culture and brain abscess culture
8	*Prevotella baroniae* brain abscess	Probable	Blood culture
9	*Staphylococcus aureus* brain abscess	Probable	Blood culture
10	*Staphylococcus aureus* brain abscess	Probable	Blood culture
11	*Streptococcus anginosus* brain abscess	Probable	Blood culture

12	Tuberculosis	Definite	TB PCR from brain abscess
13	Tuberculosis	Probable	Blood culture
14	Tuberculosis	Probable	BAL TB PCR
15	Tuberculosis	Definite	TB PCR from brain abscess
16	Tuberculosis	Definite	CSF AFB culture
17	Tuberculosis	Definite	CSF AFB culture and TB PCR
18	Tuberculosis	Probable	Sputum AFB culture
19	Tuberculosis	Definite	CSF AFB culture and TB PCR
20	*Nocardia* brain abscess	Probable	BAL culture
21	*Nocardia* brain abscess	Probable	Blood culture
22	*Nocardia* brain abscess	Probable	Lung tissue culture
23	Toxoplasmosis	Probable	Toxoplasma serology
24	Toxoplasmosis	Definite	CSF toxoplasma PCR
25	Cryptococcosis	Definite	Serum and CSF cryptococcal antigen
26	Aspergillosis	Definite	Brain abscess culture

Abbreviations: CSF: cerebrospinal fluid, PCR: polymerase chain reaction, BAL: bronchoalveolar lavage, AFB: acid fast bacilli

Supplementary Table S3. Histological diagnosis of cases of neoplastic REBLs in training/validation set and the respective method of diagnosis

	Diagnosis	Diagnostic certainty	Method of diagnosis
1	Breast carcinoma	Probable	Primary tumor biopsy
2	Breast carcinoma	Probable	Primary tumor biopsy
3	Breast carcinoma	Probable	Primary tumor biopsy
4	Breast carcinoma	Definite	Brain biopsy
5	Breast carcinoma	Probable	Primary tumor biopsy
6	Breast carcinoma	Probable	Primary tumor biopsy
7	Breast carcinoma	Probable	Primary tumor biopsy
8	Breast carcinoma	Definite	Brain biopsy
9	Breast carcinoma	Definite	Brain biopsy
10	Breast carcinoma	Definite	Brain biopsy
11	Breast carcinoma	Definite	Brain biopsy
12	Breast carcinoma	Probable	Primary tumor biopsy
13	Breast carcinoma	Probable	Primary tumor biopsy
14	Lung adenocarcinoma	Definite	Brain biopsy
15	Lung adenocarcinoma	Definite	Brain biopsy
16	Lung adenocarcinoma	Definite	Brain biopsy
17	Lung adenocarcinoma	Definite	Brain biopsy

18	Lung adenocarcinoma	Definite	Brain biopsy
19	Lung adenocarcinoma	Definite	Brain biopsy
20	Lung adenocarcinoma	Definite	Brain biopsy
21	Lung adenocarcinoma	Definite	Brain biopsy
22	Lung squamous cell carci-noma	Probable	Primary tumor biopsy
23	Lung squamous cell carci-noma	Definite	Brain biopsy
24	Small cell lung cancer	Probable	Primary tumor biopsy
25	Small cell lung cancer	Probable	Primary tumor biopsy
26	Small cell lung cancer	Probable	Primary tumor biopsy
27	Anorectal adenocarcinoma	Probable	Primary tumor biopsy
28	Caecal adenocarcinoma	Probable	Primary tumor biopsy
29	Colon adenocarcinoma	Probable	Primary tumor biopsy
30	Colorectal adenocarcinoma	Probable	Primary tumor biopsy
31	Colorectal carcinoma	Probable	Primary tumor biopsy
32	Rectal carcinoma	Probable	Primary tumor biopsy
33	Sigmoid carcinoma	Probable	Primary tumor biopsy
34	Anal adenocarcinoma	Definite	Brain biopsy

35	Diffuse large B-cell lym-phoma	Definite	Brain biopsy
36	Diffuse large B-cell lym-phoma	Definite	Brain biopsy
37	Diffuse large B-cell lym-phoma	Definite	Brain biopsy
38	Diffuse large B-cell lym-phoma	Probable	Primary tumor biopsy
39	Diffuse large B-cell lym-phoma	Definite	Brain biopsy
40	Follicular lymphoma	Probable	Primary tumor biopsy
41	Endometrial adenocarcino-ma	Definite	Brain biopsy
42	Endometrial adenocarcino-ma	Probable	Primary tumor biopsy
43	Ovarian carcinoma	Probable	Primary tumor biopsy
44	Renal cell carcinoma	Probable	Primary tumor biopsy
45	Renal cell carcinoma	Definite	Brain biopsy
46	Liposarcoma	Probable	Primary tumor biopsy
47	Sarcoma	Probable	Primary tumor biopsy
48	Carcinoma of signet ring morphology	Definite	Brain biopsy
49	Carcinoma with neuroen-docrine features	Definite	Brain biopsy
50	Melanoma	Probable	Primary tumor biopsy

51	Pancreatic adenocarcinoma	Probable	Primary tumor biopsy
52	Astrocytoma	Definite	Brain biopsy
53	Astrocytoma	Definite	Brain biopsy
54	Astrocytoma	Definite	Brain biopsy
55	Astrocytoma	Definite	Brain biopsy
56	Glioblastoma	Definite	Brain biopsy
57	Glioblastoma	Definite	Brain biopsy
58	Glioblastoma	Definite	Brain biopsy
59	Glioblastoma	Definite	Brain biopsy
60	Glioblastoma	Definite	Brain biopsy
61	Glioblastoma	Definite	Brain biopsy
62	Glioblastoma	Definite	Brain biopsy
63	Glioblastoma	Definite	Brain biopsy
64	Glioblastoma	Definite	Brain biopsy
65	Glioblastoma	Definite	Brain biopsy
66	Glioblastoma	Definite	Brain biopsy

Supplementary Table S4. Diagnoses of the cases of infective REBLs in the external holdout test set and the respective method of diagnosis

	Diagnosis	Diagnostic certainty	How diagnosis was made
1	*Aggregatibacter actinomycetem-comitans* brain abscess	Probable	Blood culture
2	Disseminated *Klebsiella pneumoniae* infection with brain abscess	Probable	Blood culture
3	Disseminated *Klebsiella pneumoniae* infection with brain abscess	Definite	Brain abscess culture
4	Polymicrobial pyogenic brain abscess	Probable	Blood culture
5	Polymicrobial pyogenic brain abscess	Definite	Brain abscess culture
6	*Enterococcus faecalis* brain abscess	Definite	Brain abscess culture
7	*Streptococcus intermedius* brain abscess	Definite	Brain abscess culture
8	*Streptococcus anginosus* brain abscess	Definite	Brain abscess culture
9	*Streptococcus anginosus* brain abscess	Definite	Brain abscess culture
10	*Pseudomonas aeruginosa* brain abscess	Probable	External auditory canal culture
11	Tuberculosis	Probable	Sputum AFB culture
12	Tuberculosis	Definite	CSF AFB culture
13	Tuberculosis	Probable	Sputum AFB culture

14	Tuberculosis	Probable	Sputum AFB culture
15	Tuberculosis	Probable	TB quantiferon and response to treatment
16	Tuberculosis	Definite	CSF AFB culture
17	Tuberculosis	Probable	Sputum AFB culture
18	Tuberculosis	Probable	Sputum AFB culture
19	*Nocardia* brain abscess	Definite	Brain abscess culture
20	Toxoplasmosis	Definite	CSF toxoplasma PCR
21	Toxoplasmosis	Definite	Brain tissue toxoplasma PCR
22	Toxoplasmosis	Definite	CSF toxoplasma PCR
23	Toxoplasmosis	Probable	Toxoplasma serology
24	Aspergillosis	Definite	CSF galactomannan antigen
25	Cryptococcosis	Probable	Serum cryptococcal antigen

Abbreviations: AFB: acid fast bacilli, CSF: cerebrospinal fluid, PCR: polymerase chain reaction, TB: tuberculosis, NSCLC: non-small cell lung cancer

Supplementary Table S5. Diagnoses of the cases of neoplastic REBLs in the external holdout test set and the respective method of diagnosis

	Diagnosis	Diagnostic certain-ty	How diagnosis was made
1	Breast carcinoma	Probable	Primary tumor biopsy
2	Breast carcinoma	Probable	Primary tumor biopsy
3	Breast carcinoma	Probable	Primary tumor biopsy
4	Breast carcinoma	Probable	Primary tumor biopsy
5	Breast carcinoma	Probable	Primary tumor biopsy
6	Breast carcinoma	Probable	Primary tumor biopsy
7	Breast carcinoma	Probable	Primary tumor biopsy
8	Lung adenocarcinoma	Probable	Primary tumor biopsy
9	Lung adenocarcinoma	Probable	Primary tumor biopsy
10	Lung adenocarcinoma	Probable	Primary tumor biopsy
11	Lung adenocarcinoma	Probable	Primary tumor biopsy
12	Lung squamous cell carcinoma	Probable	Primary tumor biopsy
13	Non-small cell lung cancer	Probable	Primary tumor biopsy
14	Non-small cell lung cancer	Probable	Primary tumor biopsy

15	Small cell lung cancer	Definite	Brain biopsy
16	Rectal carcinoma	Probable	Primary tumor biopsy
17	Sigmoid adenocarcinoma	Definite	Brain biopsy
18	Sigmoid adenocarcinoma	Probable	Primary tumor biopsy
19	Diffuse large B-cell lymphoma	Definite	Brain biopsy
20	Diffuse large B-cell lymphoma	Definite	Brain biopsy
21	Renal cell carcinoma	Probable	Primary tumor biopsy
22	Adenocarcinoma of unknown primary	Probable	Primary tumor biopsy
23	Melanoma	Probable	Primary tumor biopsy
24	Germ cell tumour	Definite	Brain biopsy
25	Astrocytoma	Definite	Brain biopsy
26	High grade glioma	Definite	Brain biopsy
27	Glioblastoma	Definite	Brain biopsy
28	Glioblastoma	Definite	Brain biopsy
29	Glioblastoma	Definite	Brain biopsy
30	Glioblastoma	Definite	Brain biopsy
31	Glioblastoma	Definite	Brain biopsy
32	Glioblastoma	Definite	Brain biopsy

Supplementary Figure S1. Confusion matrices of the MLP-based radiomics models across feature groups in the cross-validation and independent test set.

(a) Cross-validation set (mean ± SE, 95% CI)

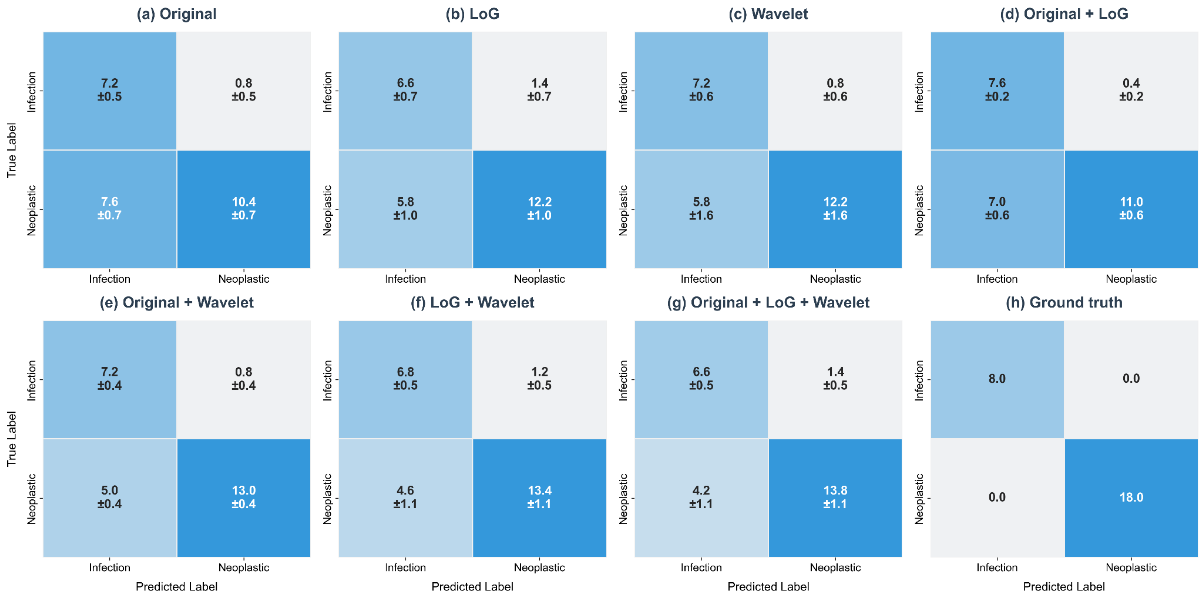

(b) Test set

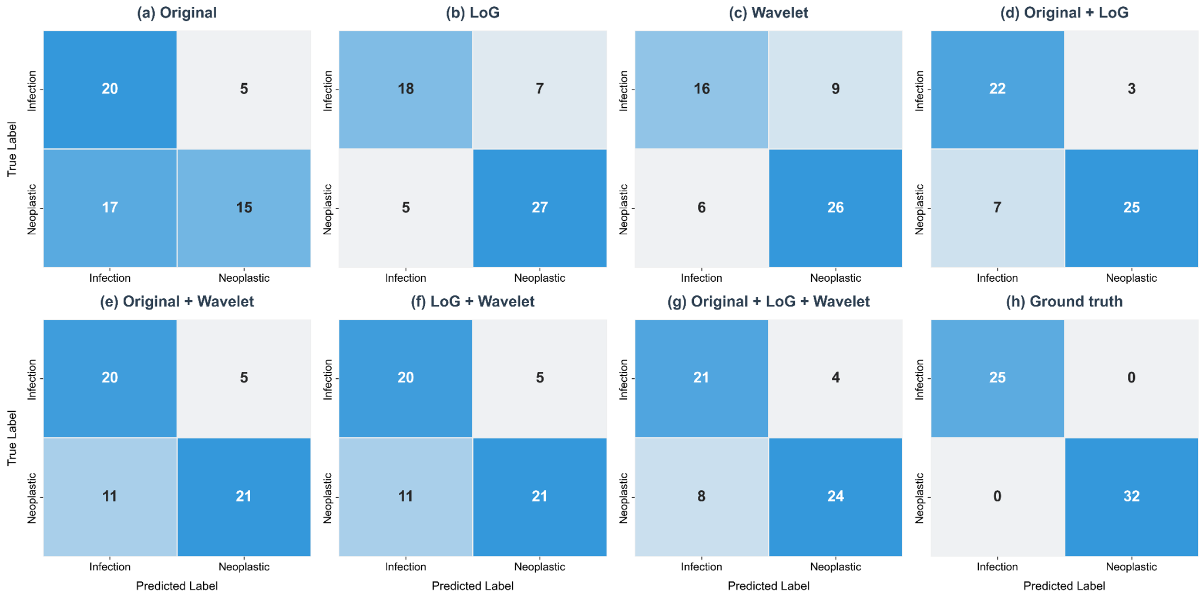

Supplementary Table S6. Error analysis of false negative and false positive cases for the MLP-based radiomics models on the independent test set across radiomic feature groups

(a) False Negative (FN)

Diagnosis	O	L	W	O+W	L+W	O+L	O+L+W	Total
Polymicrobial pyogenic brain abscess	1	1	0	0	1	0	1	4
Polymicrobial pyogenic brain abscess	0	0	0	0	1	0	0	1
Polymicrobial pyogenic brain abscess	0	0	1	0	0	0	0	1
Disseminated *Klebsiella pneumoniae* infection with brain abscess	1	1	0	1	0	0	0	3
Disseminated *Klebsiella pneumoniae* infection with brain abscess	0	0	1	0	0	0	0	1
Aggregatibacter actinomycetemcomitans brain abscess	1	1	1	1	1	0	1	6
Tuberculosis	1	1	1	0	0	0	1	4
Tuberculosis	1	0	1	0	0	0	0	2
Tuberculosis	0	0	1	0	0	0	1	2
Tuberculosis	0	0	0	1	0	0	0	1
Tuberculosis	0	0	0	1	0	0	0	1
Tuberculosis	0	0	0	0	1	0	0	1
Tuberculosis	0	0	1	0	0	0	0	1
Cerebral toxoplasmosis	0	1	1	1	0	1	0	4
Cerebral toxoplasmosis	0	0	0	0	1	1	0	2
Cerebral toxoplasmosis	0	1	0	0	0	0	0	1
Cerebral toxoplasmosis	0	0	1	0	0	0	0	1

Nocardia brain abscess	0	1	0	0	0	1	0	2
Total	5	7	9	5	5	3	4	38

(b) False Positive (FP)

**Diagnosis**	**O**	**L**	**W**	**O+W**	**L+W**	**O+L**	**O+L+W**	**Total**
Metastatic breast carcinoma	1	0	0	1	1	1	1	5
Metastatic lung adenocarcinoma	1	0	0	1	1	0	1	4
Metastatic non-small cell lung cancer	1	0	1	0	1	0	1	4
Metastatic breast carcinoma	0	1	1	1	0	0	0	3
Metastatic sigmoid adenocarcinoma	1	0	0	1	0	0	1	3
Metastatic breast carcinoma	1	0	0	0	0	0	1	2
Metastatic breast carcinoma	1	0	0	1	0	0	0	2
Metastatic lung adenocarcinoma	0	0	0	0	0	1	1	2
Metastatic lung squamous cell carcinoma	0	0	1	1	0	0	0	2
Metastatic sigmoid carcinoma	1	0	0	0	0	1	0	2
Metastatic renal cell carcinoma	1	0	0	0	0	1	0	2
Metastatic melanoma	0	0	0	0	1	1	0	2
Metastatic adenocarcinoma of unknown primary	0	0	0	0	1	1	0	2
Metastatic breast carcinoma	0	0	0	1	0	0	0	1
Metastatic breast carcinoma	0	0	0	0	1	0	0	1
Metastatic breast carcinoma	1	0	0	0	0	0	0	1
Metastatic lung adenocarcinoma	0	0	1	0	0	0	0	1
Metastatic lung adenocarcinoma	0	0	1	0	0	0	0	1
Small cell lung cancer	1	0	0	0	0	0	0	1
Metastatic rectal carcinoma	0	0	0	1	0	0	0	1
Glioblastoma	1	1	0	1	1	1	0	5

Glioblastoma	1	0	1	1	1	0	0	4
High grade glioma	0	0	0	0	1	0	1	2
Glioblastoma	1	1	0	0	0	0	0	2
Glioblastoma	1	0	0	0	0	0	0	1
Glioblastoma	1	0	0	0	0	0	0	1
Glioblastoma	0	0	0	1	0	0	0	1
Astrocytoma	1	0	0	0	0	0	0	1
Diffuse large B cell lymphoma	1	1	0	0	1	0	1	4
Diffuse large B cell lymphoma	0	1	0	0	1	0	0	2
Total	17	5	6	11	11	7	8	65

Supplementary Table S7. Distribution of retained radiomic features by category (shape, intensity and textural) across different filter groups.

Filter Group	Shape	Intensity	Textural	Total
Original	2	1	11	14
LoG	N/A	14	33	47
Wavelet	N/A	18	43	61
Original+LoG	2	15	44	61
Original+Wavelet	2	19	54	75
LoG+Wavelet	N/A	32	76	108
Original+LoG+Wavelet	3	33	87	123

The table summarizes the number of features retained after mutual information (MI) selection according to radiomic feature category and image filter group. Textural features include those derived from GLCM, GLRLM, GLSZM, GLDM, and NGTDM matrices. "N/A" indicates that shape features were not generated for filtered images (LoG, Wavelet).

List of features most frequently retained across different filter groups:

- Shape: Maximum2DDiameterColumn; SurfaceVolumeRatio
- Intensity: RootMeanSquared; Median; Skewness; 10Percentile; MeanAbsoluteDeviation; Mean
- GLCM: Contrast; Autocorrelation; JointAverage; ClusterProminence; SumEntropy; DifferenceEntropy; ClusterShade.
- GLRLM: HighGrayLevelRunEmphasis; LowGrayLevelRunEmphasis; LongRunEmphasis; LongRunHighGrayLevelEmphasis.
- GLSZM: SizeZoneNonUniformityNormalized; SmallAreaEmphasis.
- GLDM: LowGrayLevelEmphasis; HighGrayLevelEmphasis.
- NGTDM: Complexity; Strength.
